# First Report of a Whole-Genome Shotgun Sequence of a Clinical *Enterococcus faecalis* Sequence Type 6 Strain from South Africa

**DOI:** 10.1128/genomeA.01382-17

**Published:** 2017-12-14

**Authors:** Nontombi Marylucy Mbelle, Nontuthuko Excellent Maningi, Vhudzani Tshisevhe, Lesedi Modipane, Daniel Gyamfi Amoako, John Osei Sekyere

**Affiliations:** a Department of Medical Microbiology, Faculty of Health Sciences, University of Pretoria, Pretoria, South Africa; b National Health Laboratory Service, Pretoria, South Africa; c Biomedical Resource Unit, School of Laboratory Medicine and Medical Sciences, University of KwaZulu-Natal, Durban, South Africa; d Department of Pharmaceutics, Faculty of Pharmacy & Pharmaceutical Sciences, Kwame Nkrumah University of Science and Technology, Kumasi, Ghana

## Abstract

*Enterococcus faecalis* is a lactic acid-producing Gram-positive bacterium commonly found in the intestinal tract of humans and animals; it is implicated in multidrug-resistant nosocomial infections. The draft genome of this *E. faecalis* sequence type 6 (ST6) strain consists of 3,215,228 bp, with 37.20% GC content, 3,048 predicted coding sequences, and 61 RNA genes.

## GENOME ANNOUNCEMENT

*Enterococcus faecalis* is part of the human microbiota and is implicated in several fatal clinical infections, such as bacteremia and infective endocarditis (1). To our knowledge, this is the first draft genome sequence of a clinical *E. faecalis* strain, ST-6:CF006, from South Africa and the rest of Africa. This vancomycin-susceptible sequence type 6 (ST6) strain was isolated from the urine of a 41-year-old male patient hospitalized in Kalafong Hospital in Pretoria, South Africa, in 2013.

The strain was grown overnight anaerobically at 37°C in brain heart infusion (BHI) broth (Oxoid, UK) and was catalase negative but esculin hydrolysis and pyrrolidonyl arylamidase (PYR) positive. The identification was confirmed with Vitek 2 (bioMérieux, France). Genomic DNA was sheared to 200-bp libraries; 280-bp fragments were selected using 2% agarose gels and Pippin Prep (Sage Science, USA). Individual libraries were pooled to 100 pM and sequenced on the Ion Proton (Thermo, Fisher, USA) at a coverage of 89.84×. The raw reads were *de novo* assembled using the SPAdes assembler (2).

The size, GC content, number of contigs, *N*_50_, and *L*_50_ of the draft genome were 3,215,228 bp, 37.20%, 198, 104,004 bp, and 10 bp, respectively. Annotation with Rapid Annotations using Subsystems Technology (RAST) (3) and prokaryotic genome annotation pipeline (PGAP) (4) resulted in 3,048 protein-coding genes, 376 (10.98%) hypothetical proteins, 57 tRNAs, 3 rRNAs, and 4 noncoding RNAs. CRISPRFinder (5) predicted two clustered regularly interspaced short palindromic repeat 1 (CRISPR1) arrays each on nodes/contigs 3 and 59.

BLASTN analysis showed ST-6:CF006 to be closely related to the following *E. faecalis* strains with 99% nucleotide identity: V583 (GenBank accession no. AE016830), a clinical isolate from the United States; sorialis (accession no. CP015883), a fecal isolate from the United States; DD14 (accession no. CP021161), a meconium isolate from France; and L12 (accession no. CP018102) from swine in Brazil.

Resistome annotation with GoSeqIt and ResFinder (6, 7) showed aminoglycoside [*aph(3′)-III, ant(6)-Ia, aac(6′)-aph(2′′)*], macrolide-lincosamide-streptogramin (*isaA* and* mphD*), and tetracycline (*tetM*) resistance genes. ST-6:CF006 was resistant (R) to gentamicin, streptomycin, erythromycin (R > 8 µg/ml), clindamycin (R > 8 µg/ml), tetracycline (R > 168 µg/ml), ciprofloxacin (R > 88 µg/ml), and moxifloxacin (R > 88 µg/ml) but susceptible to ampicillin (>2 µg/ml), teicoplanin (≤0.5 µg/ml), linezolid (2 µg/ml), tigecycline (≤0.12 µg/ml), and vancomycin (1 µg/ml), in agreement with the resistome annotation. As no ﬂuoroquinolone resistance genes were identiﬁed, mutations in a chromosome-borne DNA gyrase gene (*gyrA*) (8, 9) were further investigated using tBLASTn; fluoroquinolone-susceptible *E. faecalis* ATCC 29212 (accession no. CP008816) was used as the reference/wild-type strain to call single-nucleotide polymorphisms (SNPs). A Ser84Ile mutation, previously implicated in fluoroquinolone resistance (8), was identified. Four plasmid replicon types, i.e., *rep2*, *rep6*, *rep9*, and *repUS11*, were identified with PlasmidFinder version 1.3 (10). The GoSeqIt VirulenceFinder database (6) discovered 19 virulence factor genes: collagen adhesion (*ace*), pheromone precursor lipoproteins (*cad, camE, cCF10*, and* cOB1*), cytolysin (*cylA, cylB, cylL,* and* cylM*), endocarditis- and bioﬁlm-associated pili (*ebpA, ebpB,* and* ebpC*), endocarditis antigen A (*efaAfs*), enterococcal leucine-rich internalin-like protein A (*elrA*), gelatinase (*gelE*), hyaluronidase (*hylA* and* hylB*), sortase A (*srtA*), and thiolperoxidase (*tpx*), contributing to its ability to aggregate, adhere, lyse, and invade host tissues. Analysis of the genomes of *E. faecalis* will increase our insight into the factors that mediate its pathogenesis and antibiotic resistance, as well as establish a correlation between genomic and phenotypic data.

### Accession number(s).

This whole-genome shotgun project has been submitted to the National Center for Biotechnology Information GenBank database under the accession no. NXKG00000000. The version described in this paper is version NXKG01000000.

## References

[1] Van Tyne D, Martin MJ, Gilmore MS 2013 Structure, function, and biology of the *Enterococcus faecalis* cytolysin. Toxins 5:895–911. doi:10.3390/toxins5050895. 23628786 PMC3709268

[2] Bankevich A, Nurk S, Antipov D, Gurevich AA, Dvorkin M, Kulikov AS, Lesin VM, Nikolenko SI, Pham S, Prjibelski AD, Pyshkin AV, Sirotkin AV, Vyahhi N, Tesler G, Alekseyev MA, Pevzner PA 2012 SPAdes: a new genome assembly algorithm and its applications to single-cell sequencing. J Comput Biol 19:455–477. doi:10.1089/cmb.2012.0021. 22506599 PMC3342519

[3] Aziz RK, Bartels D, Best AA, Dejongh M, Disz T, Edwards RA, Formsma K, Gerdes S, Glass EM, Kubal M, Meyer F, Olsen GJ, Olson R, Osterman AL, Overbeek RA, McNeil LK, Paarmann D, Paczian T, Parrello B, Pusch GD, Reich C, Stevens R, Vassieva O, Vonstein V, Wilke A, Zagnitko O 2008 The RAST server: rapid annotations using subsystems technology. BMC Genomics 9:75. doi:10.1186/1471-2164-9-75. 18261238 PMC2265698

[4] Tatusova T, DiCuccio M, Badretdin A, Chetvernin V, Nawrocki EP, Zaslavsky L, Lomsadze A, Pruitt KD, Borodovsky M, Ostell J 2015 NCBI prokaryotic genome annotation pipeline. Nucleic Acids Res 43:D599–D605. doi:10.1093/nar/gku1062. 27342282 PMC5001611

[5] Grissa I, Vergnaud G, Pourcel C 2007 CRISPRFinder: a web tool to identify clustered regularly interspaced short palindromic repeats. Nucleic Acids Res 35:W52–W57. doi:10.1093/nar/gkm360. 17537822 PMC1933234

[6] Thomsen MC, Ahrenfeldt J, Cisneros JL, Jurtz V, Larsen MV, Hasman H, Aarestrup FM, Lund O 2016 A bacterial analysis platform: an integrated system for analysing bacterial whole genome sequencing data for clinical diagnostics and surveillance. PLoS One 11:e0157718. doi:10.1371/journal.pone.0157718. 27327771 PMC4915688

[7] Zankari E, Hasman H, Cosentino S, Vestergaard M, Rasmussen S, Lund O, Aarestrup FM, Larsen MV 2012 Identiﬁcation of acquired antimicrobial resistance genes. J Antimicrob Chemother 67:2640–2644. doi:10.1093/jac/dks261. 22782487 PMC3468078

[8] Aldred KJ, Kerns RJ, Osheroff N 2014 Mechanism of quinolone action and resistance. Biochemistry 53:1565–1574. doi:10.1021/bi5000564. 24576155 PMC3985860

[9] Osei Sekyere J, Amoako DG 2017 Genomic and phenotypic characterisation of fluoroquinolone resistance mechanisms in *Enterobacteriaceae* in Durban, South Africa. PLoS One 12:e0178888. doi:10.1371/journal.pone.0178888. 28636609 PMC5479536

[10] Carattoli A, Zankari E, García-Fernández A, Voldby Larsen M, Lund O, Villa L, Møller Aarestrup F, Hasman H 2014 *In silico* detection and typing of plasmids using PlasmidFinder and plasmid multilocus sequence typing. Antimicrob Agents Chemother 58:3895–3903. doi:10.1128/AAC.02412-14. 24777092 PMC4068535

